# Carbenoxolone-sensitive and cesium-permeable potassium channel in the rod cells of frog taste discs

**DOI:** 10.1016/j.bbrep.2015.09.010

**Published:** 2015-09-24

**Authors:** Yukio Okada, Toshihiro Miyazaki, Rie Fujiyama, Kazuo Toda

**Affiliations:** a Integrative Sensory Physiology, Graduate School of Biomedical Sciences, Nagasaki University, Nagasaki 852-8588, Japan; b Cell Biology, Graduate School of Biomedical Sciences, Nagasaki University, Nagasaki 852-8588, Japan

**Keywords:** Taste disc, Rod cell, Voltage-gated K^+^ channel, Cs^+^-permeable, Carbenoxolone, Lithobates catesbeianus

## Abstract

The rod cells in frog taste discs display the outward current and maintain the negative resting potential in the condition where internal K^+^ is replaced with Cs^+^. We analyzed the properties of the Cs^+^-permeable conductance in the rod cells. The current–voltage (*I/V*) relationships obtained by a voltage ramp were bell-shaped under Cs^+^ internal solution. The steady state *I/V* relationships elicited by voltage steps also displayed the bell-shaped outward current. The activation of the current accelerated with the depolarization and the inactivation appeared at positive voltage. The gating for the current was maintained even at symmetric condition (Cs^+^ external and internal solutions). The wing cells did not show the properties. The permeability for K^+^ was a little larger than that for Cs^+^. Internal Na^+^ and NMDG^+^ could not induce the bell-shaped outward current. Carbenoxolone inhibited the bell-shaped outward Cs^+^ current dose dependently (*IC*_*50*_: 27 μM). Internal arachidonic acid (20 μM) did not induce the linear current–voltage (*I–V*) relationship which is observed in two-pore domain K^+^ channel (K_2P_). The results suggest that the resting membrane potentials in the rod cells are maintained by the voltage-gated K^+^ channels.

## Highlights

• Frog taste cells displayed bell-shaped outward current under Cs^+^ internal solution.
• The gating for the current was maintained even at symmetric ionic condition.
• Carbenoxolone inhibited the outward Cs^+^ current dose dependently (*IC*_*50*_: 27 μM).
• The resting potentials may be maintained by the voltage-gated K^+^ channels.

## 1 Introduction

The resting membrane potentials in animal cells are maintained with a potassium permeability of the plasma membrane. In mammals, the resting potassium conductance could be simply explained by a background (leak) K^+^ current conducted through potassium-selective pores in the plasma membrane [1]. The background K^+^ current can conduct through a voltage-gated K^+^ channel (Kv), inward rectifying K^+^ channel (Kir) or two-pore domain K^+^ channel (K_2P_) [2].

Mammalian taste cells express pH-sensitive two-pore domain K^+^ channels, suggesting the contribution of the channels to resting potential and sour taste perception [3], [4]. Relatively large cells (wing cells) in frog taste discs displayed extracellular K^+^- and H^+^-sensitive K^+^ conductance [5], while small spindle-type cells (rod cells) showed H^+^-activated outward K^+^ current [6]. The rod cells also displayed a Cs^+^-permeable outward current with Cs^+^ internal solution [7]. In the present study, we analyzed the properties of the Cs^+^-permeable conductance in frog rod cells.

## 2 Materials and methods

### 2.1 Cell preparation

Adult bullfrogs (*Lithobates catesbeianus*) weighing 250–550 g were used for the experiment over the course of a year. Experiments were performed in accordance with the Guidelines for Animal Experimentation of Nagasaki University with approval of the Institutional Animal Care and Use Committee. The keeping of bullfrogs (invasive alien species) was approved by the Ministry of the Environment of Japan (approval number 06000204). Taste disc cells were isolated from the tongue of decapitated and pithed animals, as described previously [8]. Fungiform papillae were dissected from the tongue in nominal Ca^2+^-free saline and stored in Ca^2+^-free saline containing 2 mM EDTA (ethylenediaminetetraacetic acid) for 10 min. These papillae were bathed in the same saline containing 10 mM l-cysteine and 10 U/ml papain (Sigma, St Lois, MO, USA) for 10–13 min. The papillae were then rinsed with normal saline, and individual cells were dissociated by gentle trituration in normal saline. Isolated taste disc cells showing a characteristic morphology were readily distinguished from the other cells and classified into rod and wing cells [8]. Rod cells had one dendrite-like process while wing cells had two or three dendrite-like processes connected to each other by a sheet-like structure.

### 2.2 Electrophysiological recording

Voltage-clamp recording was performed in the whole-cell configuration [9] using an EPC-7 plus amplifier (HEKA Elektronik, Lambrecht, Germany). Patch pipettes were pulled from Pyrex glass capillaries containing a fine filament (Ken Enterprise, Kanagawa, Japan) with a two-stage puller (Narishige PD-5, Tokyo, Japan). The tips of the electrodes were heat-polished with a microforge (Narishige MF-80). The resistance of the resulting patch electrode was 5–10 MΩ when filled with internal solution. The formation of 5–20 GΩ seals between the patch pipette and cell surface was facilitated by applying weak suction to the interior of the pipette. The patch membrane was broken by applying strong suction, resulting in a sudden increase in capacitance. Recordings were made from taste disc cells that had been allowed to settle on the bottom of a chamber placed on the stage of an inverted microscope (Olympus IMT-2, Tokyo, Japan). The recording pipette was positioned with a hydraulic micromanipulator (Narishige WR-88). We never used a material to make the cells stick firmly to the bottom of the chamber. The cells were picked up by the patch pipette a little. The current signal was low-pass-filtered at 3 kHz, digitized at 125 kHz using a TL-1 interface (Axon Instruments, Union City, CA, USA), acquired at a sampling rate of 0.25–5 kHz using a computer running the pCLAMP 5.5 software (Axon Instruments), and stored on a hard disk. pCLAMP was also used to control the digital-analogue converter for the generation of the clamp protocol. The indifferent electrode was a chloriding silver wire. Voltages were corrected for the liquid junction potential between the external and internal solutions. Capacitance and series resistance were compensated for, as appropriate. In voltage ramp-mode experiments, the voltage was held at −50 mV, which was close to the resting potential. The value of −50 mV was compensated to −55 mV when Cs^+^ internal solution was used for the correction of the liquid junction potential. The whole-cell current–voltage (*I/V*) relationship was obtained from the current generated by the 167 mV/s voltage ramp from −100 to +100 mV. In some case, the current–voltage relationships were obtained from the currents generated by the 80 ms voltage-step pulses between −105 and +55 mV in 10 mV increments from a holding potential of −85 mV. The voltage in step pulses also was compensated for the liquid junction potential. Input resistance was calculated from the slope conductance generated by the voltage ramp from–100 to–50 mV. Data were analyzed with pCLAMP and Origin 2015 (Origin Lab, Northampton, MA, USA). Unless stated otherwise, the data are presented as means±SEM, with significance being tested by the Student's t test. Differences were considered significant if *P*<0.05.

### 2.3 Solutions and drugs

Control (normal) solution consisted of (in mM): NaCl, 115; KCl, 2.5; CaCl_2_, 1.8; Hepes (4–(2-hydroxyethyl)-1-piperazineethanesulfonic acid), 10; glucose, 20; pH 7.2. The pH of normal saline and other solutions was adjusted by Tris (tris(hydroxymethyl)aminomethane) base. K^+^ and Cs^+^ saline solutions were prepared by the replacement of Na^+^ and K^+^ in normal saline solution with K^+^ or Cs^+^. Carbenoxolone (CBX, 3–100 μM, Sigma) and Ba^2+^ (1 mM) were directly dissolved in normal saline solutions. The solution exchange was done by gravity flow. The Cs^+^ internal solution contained (in mM): CsCl, 100; CaCl_2_, 0.1; MgCl_2_, 2, EGTA, 1; Hepes, 10; pH 7.2. In some experiments, internal Cs^+^ was replaced with Na^+^ or NMDG^+^. For stock solution, arachidonic acid (20 mM, Cayman, Ann Arbor, MI, USA) was dissolved in dimethylsulfoxide (DMSO). Samples of the stock solutions were added to internal solution to give the desired final solution.

All experiments were carried out at room temperature (20–25 °C).

## 3 Results and discussion

### 3.1 Cs^+^ current in the rod cells in frog taste disc

**Fig. 1:**
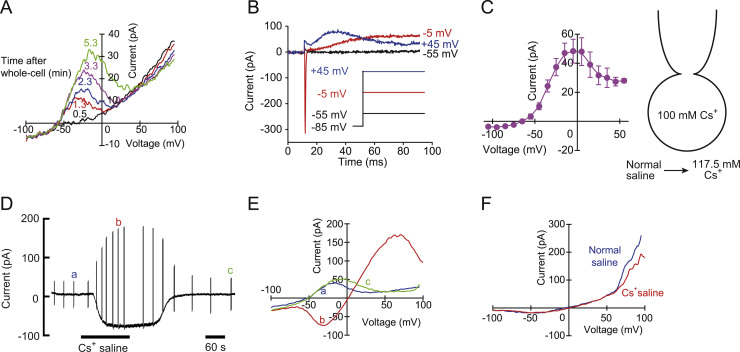
Effects of internal Cs^+^ on the membrane properties of the rod and wing cells in frog taste discs. (A) Time course of the whole-cell current–voltage (*I/V*) relationships elicited by the voltage ramp (167 mV/s) from −100 mV to +100 mV after whole-cell attainment with Cs^+^ internal solution in a rod cell. (B) The currents elicited by 80 ms voltage steps to −55, −5 and +45 mV from a holding potential of −85 mV in normal saline solution. The initial transient inward current at −5 mV was voltage-gated Na^+^ current. Leak currents were not subtracted from the current traces. (C) Current–voltage (*I/V*) relationships for the currents measured at the end of the pulse. Raw current values were plotted. Values are mean±SEM from 3 cells. (D) Pen recording of the current signal at a holding potential of −55 mV while external normal saline solution was replaced with Cs^+^ saline solution in the rod cell. All transient current deflections were elicited by the voltage ramp. (E) Plots of the whole-cell *I/V* relationships elicited by the voltage ramp in the rod cell. The relationships labeled as a, b and c were obtained at the times indicated by the same letters on the pen recording. (F) Plots of the whole-cell *I–V* relationships elicited by the voltage ramp in a wing cell.

As reported previously [7], the rod cells in frog taste discs with K^+^ internal solution displayed the mean resting potential of −49 mV and s-shaped current–voltage relationships evoked by a voltage ramp between −100 mV and +100 mV in normal saline solution. Under Cs^+^ internal solution, frog rod cells displayed a resting potential of −48.9±1.1 mV (*n*=38). Therefore, the replacement of internal K^+^ with Cs^+^ did not change the resting potential. The current–voltage (*I/V*) relationships obtained by a voltage ramp were bell-shaped under Cs^+^ internal solution (Fig. 1A). The bell-shaped current appeared within 2 min after whole-cell attainment. The steady state *I/V* relationships elicited by voltage steps also displayed the bell-shaped outward current (Fig. 1C) (*n*=3). The activation of the current accelerated with the depolarization and the inactivation appeared at positive voltage (Fig. 1B and C). Interestingly, frog wing cells showed the bell-shaped current–voltage relationships with K^+^ internal solution [10]. The wing cells with Cs^+^ internal solution in normal saline solution showed a novel small inward current at negative voltage (Fig. 1F) and the current reversed at the membrane potential of +5.2±0.9 mV (*n*=40) [10]. Thus, the negative resting potential disappeared in the wing cells with Cs^+^ internal solution. The basal properties of wing and rod cells were summarized in Table 1.

**Table 1:** Basal properties of wing (Ib) and rod (II) cells in a condition of control (normal) external and Cs^+^ internal solutions.

Cell type	Membrane capacitance (pF)	Resting potential (mV)	Current density at 0 mV (pA/pF)	*I–V* relationship
Wing (Ib) cells (*n*=40)	13.5±0.2	5.2±0.9	−0.19±0.03	Novel small inward current
Rod (II) cells (*n*=38)	5.6±0.2	-48.9±1.1	3.9±0.4	Bell-shaped outward current

Data are presented as means±SEM.

**Fig. 2:**
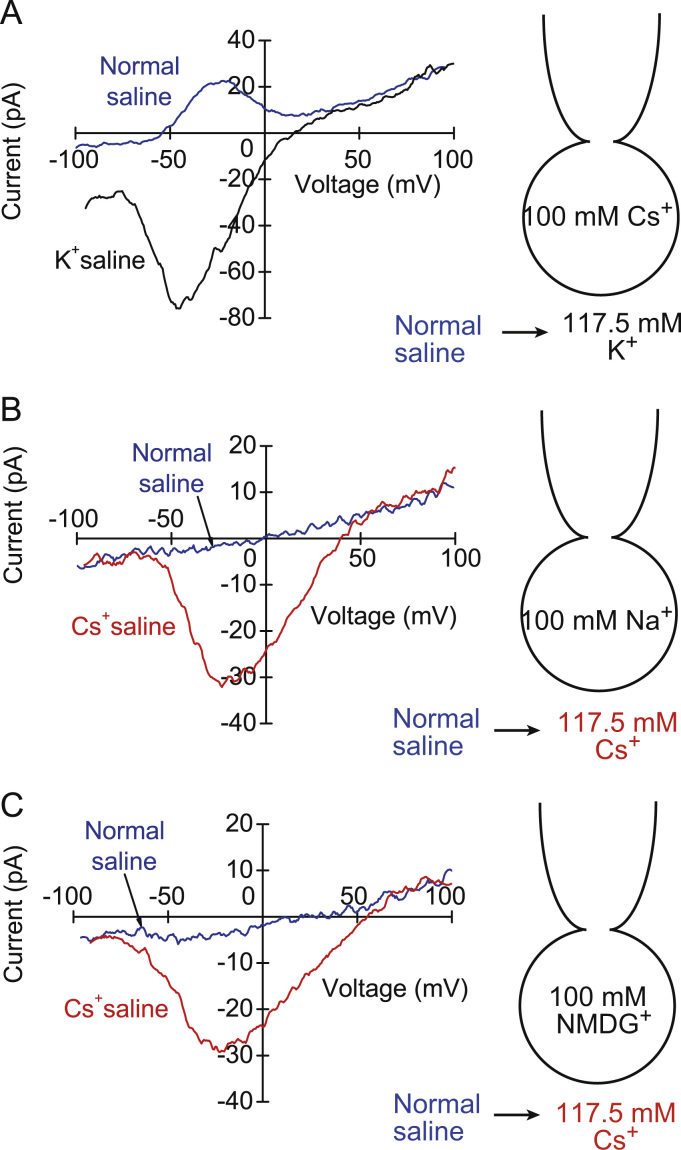
Effects of external K^+^ (A), internal Na^+^ (B) and internal NMDG^+^ (C) on the current–voltage (*I/V*) relationships in the rod cells of frog taste discs.

When external normal saline solution was replaced with Cs^+^ saline solution, the rod cells displayed an apparent inward current at the membrane potential of −55 mV (Fig. 1D). The wash-out recovered the bell-shaped outward current completely. The current–voltage relationships in both conditions of normal and Cs^+^ saline solutions indicate the consistent voltage-dependency for the currents (Fig. 1E). The current reversed at the membrane potential of +2.4±0.3 mV (*n*=10) under internal 100 mM Cs^+^ and external 117.5 mM Cs^+^ (Equilibrium potential for Cs^+^: +4 mV). External K^+^ solution also inverted the bell-shaped outward current to the inward current and the current reversed at the membrane potential of +17.0±3.1 mV (*n*=3) (Fig. 2A). The bell-shaped outward current disappeared by the replacement of internal Cs^+^ with Na^+^, while external Cs^+^ solution induced the voltage-dependent inward current (Fig. 2B). The current reversed at the membrane potential of +35.6±1.6 mV (*n*=5). Under internal NMDG^+^, the inward Cs^+^ current reversed at the membrane potential of +51.7±6.0 mV (*n*=3) (Fig. 2C). Permeability ratios relative to Cs^+^ were calculated from the reversal potentials by using the Goldman-Hodgkin-Katz equation:

(1) $$\sum P_{X}{Z_{X}}^{2}\frac{ErevF^{2}}{RT}\frac{\left[X_{O}\right]-{\left[X\right]}_{i}\exp\left(Z_{X}FE_{\mathrm{rev}}/RT\right)}{1\;-\exp(Z_{X}FE\mathrm{rev}/RT)}=0$$

where *F, R* and *T* are physical constants, Z_X_ is the valence and *P*_*X*_ is the permeability coefficient of the ion *X*, [*X*]_o_ and [*X*]_i_ are extracellular and intracellular concentrations of *X* and *E*_rev_ denotes the reversal potential. When divalent cation and anion permeabilities are ignored, the relative permeabilities were *P*_*K*_*:P*_*Cs*_*:P*_*Na*_*:P*_*NMDG*_=1.67:1:0.29:0.15.

### 3.2 Block and modulation of Cs^+^ current

**Fig. 3:**
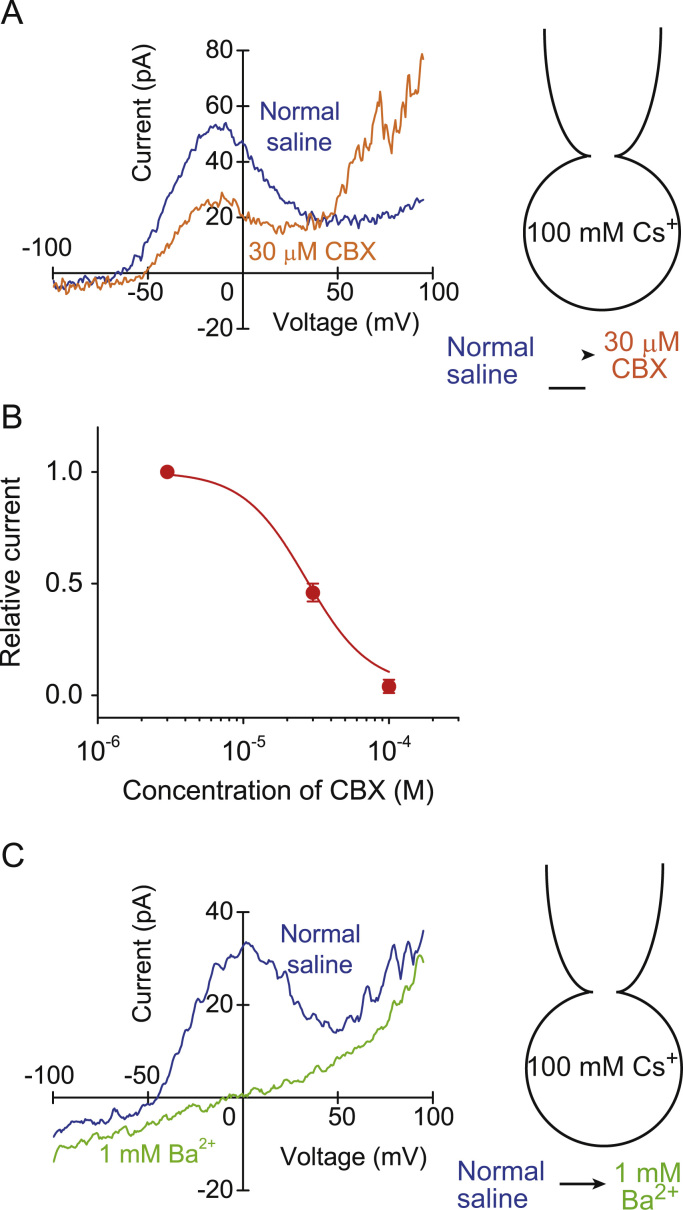
Effects of carbenoxolone (CBX) and Ba^2+^ on the bell-shaped outward Cs^+^ current in the rod cells. (A) Inhibition of the outward Cs^+^ current by 30 μM CBX. (B) Dose-response relationship of the current inhibition by CBX. Data were plotted by the Hill equation (Hill coefficient: 2). The currents at −10 mV were measured. Values are mean±SEM from 3 cells. (C) Inhibition of the outward Cs^+^ current by 1 mM Ba^2+^.

**Fig. 4:**
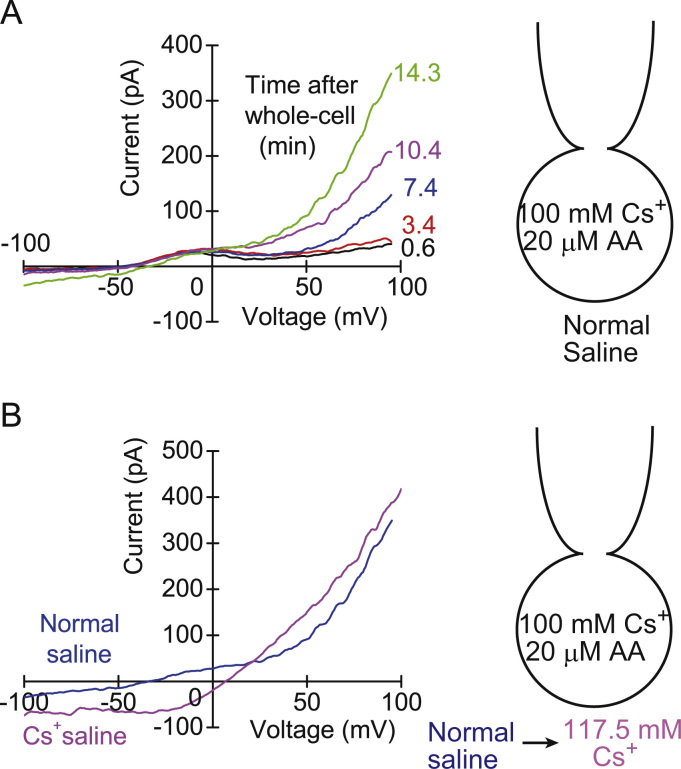
Effect of internal 20 μM arachidonic acid (AA) on the membrane properties in a rod cell. (A) Time course of the change of the current–voltage (*I/V*) relationships in the rod cell. (B) Effect of replacement of external Na^+^ and K^+^ with Cs^+^ at the steady state of internal dialysis of AA in the same cell.

The relative permeabilites calculated above suggest that the Cs^+^ permeability is not so selective. In mammalian type-II taste cells, the voltage-gated calcium homeostasis modulator 1 (CALHM1) has been identified as a channel which is indispensable for taste-stimuli-evoked ATP release [11]. The pore size is large, but the channel is insensitive to the blockers for hemichannels [11]. Carbenoxolone (CBX) is known as a specific blocker for a hemichannel, pannexin 1 [12]. We checked the possibility that the bell-shaped outward current might arise from the current through the pannexin. The drug inhibited the bell-shaped outward Cs^+^ current dose dependently (Fig. 3A and B). *IC*_*50*_ for the inhibition was 27 μM. It also enhanced the outward current at the membrane potential over +50 mV (Fig. 3A). *IC*_*50*_ at the present study was higher than it (5 μM) for pannexin, suggesting that the bell-shaped outward current did not arise from the pannexin-mediated current. Ba^2+^ (1 mM) completely blocked the bell-shaped outward current (Fig. 3C) (*n*=4). Polyunsatured fatty acids such as arachidonic acid stimulate the background K^+^ current conducted through two pore domain K^+^ channels [13]. When arachidonic acid (20 μM) was dialyzed into the rod cells, it induced a slowly developing small inward current at −50 mV (Fig. 4A). The inward current at −50 mV increased from −0.4±0.2 pA/pF to −1.6±0.3 pA/pF (*P*<0.05, *n*=4). The inward current shifted the resting membrane potential from −45±2 mV to −31±3 mV (*P*<0.05, *n*=4). Internal arachidonic acid modified a little the voltage dependency of the bell-shaped current, but permeability for Cs^+^ was consistently larger than that for Na^+^ (Fig. 4B).

Under non-symmetric condition (external normal saline and internal Cs^+^), the rod cells displayed the bell-shaped outward current. The steady-state *I–V* relationships elicited by the step pulses also showed the bell-shape which might have arisen from inactivation at the positive membrane potential. The current started to appear at the membrane potential of about −50 mV which was equal to the resting membrane potential. The gating for the current was maintained even at symmetric condition (external Cs^+^ saline and internal Cs^+^). Similarly some voltage-gated K^+^ channels show voltage-dependent outward rectification because they open with depolarization to the voltages where outward K^+^ flux is preferred. These channels do not shift their activation voltage with *E_K_* [2]. Though the permeability for Cs^+^ in the bell-shaped current is lower than that for K^+^, the resting potentials of the rod cells in frog taste discs may be influenced by both ionic permeability and gating of the resting leak channels. The involvement of the present Cs^+^-permeable channels in the resting potential may influence the excitability of rod cells. Inactivation of the voltage-gated Na^+^ channels can occur in the membrane potential of −50 mV, resulting in the failure of spike generation. It is still unclear whether the TTX-sensitive Na^+^ spike is necessary for neurotransmitter (ATP) release from taste cells or not [11], [14].

The permeability ratios estimated from the reversal potentials for Na^+^ and NMDG^+^ are a little too high, but internal Na^+^ and NMDG^+^ did not induce the bell-shaped outward current. We could not deny the possibility of leak Na^+^ current conducted through other channels. Although carbenoxolone inhibited the bell-shaped outward Cs^+^ current dose dependently, the effect was not so specific. It also elicited conductance increase in the wing cells [10]. The two-pore domain K^+^ channels display the linear *I–V* relationships in symmetric ionic condition as well as by perfusion with arachidonic acid [13], but the Cs^+^-permeable conductance in the present study did not show the properties. Regardless of the two-pore domain K^+^ channels, arachidonic acid elicited a depolarizing conductance increase in the rod cells. Endogenous lipids such as unsaturated fatty acids and their metabolites can modulate TRP channel activation by direct binding [15], [16]. TRPM5 is a cation channel that is essential for transduction of mammalian taste [17]. Although frogs are deficient in TRPM5 gene [18], the rod cells may have an unknown TRP channel related to taste transduction.
